# Production objectives and breeding practices of urban goat and sheep keepers in West Africa: regional analysis and implications for the development of supportive breeding programs

**DOI:** 10.1186/s40064-015-1075-7

**Published:** 2015-06-19

**Authors:** Luc Hippolyte Dossa, Mamadou Sangaré, Andreas Buerkert, Eva Schlecht

**Affiliations:** Animal Husbandry in the Tropics and Subtropics, University of Kassel and Georg-August-Universität Göttingen, Steinstrasse 19, 37213 Witzenhausen, Germany; Centre International de Recherche-Développement sur l’Elevage en Zone Subhumide (CIRDES), Bobo Dioulasso, Burkina Faso; Faculté des Sciences Agronomiques, Université d’Abomey-Calavi, 03 BP 2819 Jericho, Cotonou, République du Bénin; Organic Plant Production and Agroecosystems Research in the Tropics and Subtropics, University of Kassel, Steinstrasse 19, 37213 Witzenhausen, Germany

**Keywords:** Livestock functions, Selection criteria, Small ruminants, Farmer surveys, Urban production systems

## Abstract

To better understand the relative importance of the multi-purpose functions of small ruminants for their urban owners and related breeding practices including selection criteria, we undertook a comparative analysis across the West African cities of Kano (Nigeria), Bobo Dioulasso (Burkina Faso) and Sikasso (Mali). Semi-structured questionnaires were used to collect the required information from 301 sheep farmers (100, 102 and 99 in Kano, Bobo Dioulasso and Sikasso) and 306 goat farmers (100, 99 and 107 in Kano, Bobo Dioulasso and Sikasso). Sheep and goats were kept for a variety of reasons including income generation, insurance (sale for cash to meet unexpected expenditures) and economic security (sale for cash to support foreseeable expenses), social/religious functions and prestige in ownership. The relative importance given by respondents to the different functions varied significantly (p < 0.001) across cities and between species within a city. However, irrespective of city, both species were primarily kept for their financial functions whereby sheep were perceived as having higher economic value. Although breeding practices were very similar in many respects (low practice of castration, culling and replacement strategies, uncontrolled mating, no record keeping and selection criteria), the emphasis put on each selection criteria varied across cities and between species. Irrespective of city, most of the goats were of the indigenous type while keeping crossbred animals and/or maintaining more than one genotype in the same flock was more commonly practiced by sheep keepers. This points to a higher motivation for strategic breeding among sheep than goat keepers and indicates that the former might be interested in joining carefully designed participatory flock improvement programs.

## Background

Livestock keeping in and at the periphery of major and secondary urban centers is a common feature in West African countries. Until recently this was considered as out-of-place (Hovorka 2008) and as a sign of poverty (Schiere and van der Hoek 2001). Despite being an integral component of urban agriculture (UA), it has received little attention from researchers and policy-makers compared to urban crop and/or vegetable farming. Yet, a recent study conducted in the three secondary West African cities of Kano (Nigeria), Bobo Dioulasso (Burkina Faso) and Sikasso (Mali) revealed that livestock keeping rather than crop cultivation and gardening was the most popular activity among UA practitioner households (Dossa et al. 2011). Households involved in urban livestock keeping accounted for 90% of UA households in Kano, 88% in Bobo Dioulasso and 72% in Sikasso. A species analysis of livestock holdings showed that small ruminants, that is goats and sheep, were the most common species kept across the three cities. This also holds for other densely populated semi-arid areas in West Africa where Kamuanga et al. (2008) noted a significant shift from cattle to small ruminants. A case study carried out in Maradi (Niger) showed that 61% of urban households were keeping sheep and/or goats (Ali et al. 2003). However, the two species have not received equal attention in studies on urban small ruminant production in the West African context. Respective studies (Siegmund-Schultze and Rischkowsky 2001; Touré and Ouattara 2001; Killanga et al. 2004; Rischkowsky et al. 2006; Diogo et al. 2010), which are limited in number, mainly focused on sheep, neglecting goats. They are also limited in their scope: some to just the socio-economic characteristics of the keepers, most to one city, others to the management practices and flock productivity, providing therefore a patchwork view of the prevailing production systems. Hence, although locally relevant, conclusions drawn and recommendations made in each case study may not be valid for other West African countries. For instance, Rischkowsky et al. (2006), based on a study in Maroua (Cameroun), claimed that the management practices as well as the productivity of sheep in urban areas of West Africa are similar to those found in its rural areas, while recent findings by Baah et al. (2012) in the city of Kumasi (Ghana) do not support this statement. The latter authors further observed that the production objectives of urban small ruminant keepers were different from those of their rural counterparts. Caution is therefore required when making generalizations about the West African region because of the heterogeneity of socio-economic settings and urban farming systems (Dossa et al. 2011). Beyond the broader realms of providing fresh meat for people living in and around the city, both small ruminant species are important parts of urban livelihood strategies. Yet, a clear distinction between sheep and goats needs to be made: Baah et al. (2012) showed that the two species fulfill different nonfood functions within the same urban area, suggesting that urban farmers have different trait preferences for each species.

High prevalence of diseases and parasites has been reported as major constraint, causing high mortalities and preventing the animals to express their full genetic potential which is generally considered low (Dicko et al. 2006). Various studies have shown that improved management strategies are the key entry point for improving smallholder goat and sheep production, but also that there is potential for genetic improvement through selection (Bosso et al. 2007; Kosgey and Okeyo 2007; Shrestha and Jahmy 2007). Yet, an explicit understanding of the varying nonfood functions of sheep and of goats as well as a comprehensive quantification of their keepers’ preferences for traits and breeding practices including applied selection criteria within and across urban settings is therefore indispensable for making locally and regionally valid recommendations and for establishing appropriate improvement initiatives. However these aspects have received insufficient attention in the available literature. Therefore, the objectives of the current study were to record and compare the production objectives of urban sheep and goat farmers, their trait preferences and breeding practices within and across three selected West African cities.

## Methods

### Study locations

The study was carried out from April to June 2010 in the West African cities of Kano (Nigeria), Bobo Dioulasso (Burkina Faso) and Sikasso (Mali). With a population estimated at 3,140,000 inhabitants in 2007 (UNUP 2009), Kano is the second largest city in Nigeria after Lagos. It is located in the Sudano-Sahelian zone in the northern part of the country and covers a total area of 55,000 ha (Tiffen 2001). Its semi-arid climate is characterized by a dry season (October–May) and a rainy season (June–September) with an annual rainfall of about 800 mm (Valbuena et al. 2012). It is the home of the relatively small-sized Kano Brown goat, also known as Sokoto Red or Maradi goat (Wilson 1991) and of the Yankasa sheep, a medium sized sheep (Afolayan et al. 2006) originating from the long-legged or West African Sahel type sheep (Wilson 1991). The distribution of the latter is farther North in the Sahelian zone. Bobo Dioulasso is located in the South–West of Burkina Faso and is the country’s second largest city after its capital Ouagadougou. With an estimated population of 400,000 inhabitants in 2007, it covers a land area of 13,678 ha (Commune de Bobo Dioulasso 2007). Situated in the Southern part of Mali, Sikasso is the country’s third largest city after Bamako and Segou. In 2005, It covered an area of 3,745 ha (Ministère de l’Habitat et de l’Urbanisme 2005) and had close to 200,000 inhabitants. Bobo Dioulasso and Sikasso lay both in the Southern Sudanian Savana zone, characterized by a sub-humid climate with a rainy season (May–October), a cool dry season (November–February) and a hot dry season (March–May). The average annual rainfall varies between 900 and 1,200 mm. The native sheep and goats of the region are of the Djallonke and West African dwarf types described by Wilson (1991).

### Sampling procedure and data collection

In each of the study locations, urban small ruminant farmers were randomly selected through a stratified sampling procedure and were individually interviewed. A small ruminant farmer was defined as a person who owns the animals, is involved in their maintenance and is the decision maker concerning management, selection and disposal. Small ruminant farmers identified themselves as sheep or goat farmers. A total of 301 sheep farmers (100 in Kano, 102 in Bobo Dioulasso and 99 in Sikasso) and 306 goat farmers (100 in Kano, 99 in Bobo Dioulasso and 107 in Sikasso) were interviewed using semi-structured questionnaires which consisted of open and closed questions. The questionnaires covered information on the socio-economic characteristics of the respondents, reasons for keeping sheep or goats, the animals’ functions, flock sizes and structure, breeding practices and selection criteria for replacing breeding females and males. Respondents were first asked to list the production objectives and selection criteria for breeding females and males and then to rank them from the most important (1) to the least important (n). The questionnaires were pre-tested on 10 small ruminant farmers in each city to ensure that the questions were adapted to local conditions without affecting the comparability of information across locations. The interviews were conducted in each site-specific local language by trained local enumerators.

### Data analysis

All statistical analyses were performed using IBM^®^ SPSS^®^ Statistics 20 (IBM Corp. Release 2011). Two main factors were considered: species (2 subgroups: sheep, goats) and city (3 subgroups: Kano, Bobo Dioulasso, Sikasso). Descriptive statistics were performed for all variables. Cross tabulations and Chi square (χ^2^) statistics were used to compare categorical variables within and between subgroups, while arithmetic means and rank means and their standard deviations were calculated for within- and between-subgroup comparisons of the continuous variables. Within each subgroup, comparisons of mean ranks were performed using the Friedman test, which compared the distribution of preference ranks of each animal function and of each selection criterion. Post hoc analyses were then applied using the non-parametric Wilcoxon Signed-rank test with Bonferroni’s adjustments. Within each city, comparisons between sheep and goats were done using the non-parametric Mann–Whitney U test while the non-parametric Kruskal–Wallis test followed by the Mann–Whitney U test for post hoc separation of group means and mean ranks was used for comparison between cities. A statistically significant difference was reported if the *p* value was less than 0.05.

## Results

### Ownership pattern and socio-economic characteristics of urban small ruminant keepers

Out of the total of 609 persons interviewed, 295 (48%) kept both sheep and goats, 206 (34%) kept only sheep and 108 (18%) kept only goats. The proportion of people who kept both sheep and goats was significantly higher (p < 0.001) in Kano (67%) than in Sikasso (46%) and in Bobo Dioulasso (33). Keepers of only sheep were proportionally more numerous in Bobo Dioulasso and Sikasso (46 and 37%, respectively) than in Kano (19%), while keepers of only goats represented 22% of respondents in Bobo Dioulasso against 16 and 15% in Sikasso and Kano, respectively. Small ruminant keepers in all three cities were predominantly male, but the proportion of female keepers was significantly higher (p < 0.001) in Bobo Dioulasso (28%) and Kano (17%) than in Sikasso (5%). The average age of keepers was 43 ± 12 years, but keepers in Kano were significantly (p < 0.001) younger (36 ± 9 years) than those in Bobo Dioulasso (46 ± 15 years) and Sikasso (49 ± 8 years). Within cities, no significant difference in gender and age of owners was noted between sheep and goats keepers. While overall most (73%) of the small ruminant keepers had started this activity within the last 10 years, a significantly (p < 0.001) higher proportion of respondents had started this activity less than 5 years ago in Sikasso (33%) and Bobo Dioulasso (31%) as compared to Kano (4%). The main occupation of small ruminant keepers varied significantly (p < 0.001) across cities. In Bobo Dioulasso they were mainly traders (26%), people without any other income generating activity (20%) and service providers (16%). In Sikasso, they were mainly public/civil servants (32%), traders (27%) and service providers (17%), whereas traders and services providers represented 40 and 24%, respectively, of the keepers in Kano. Crop/vegetable farmers accounted for 15, 13 and 9% of keepers in Bobo Dioulasso, Kano and Sikasso, respectively. Only 9% of small ruminant keepers in Bobo Dioulasso against 1.5 and 2.4% in Kano and Sikasso considered livestock keeping as their main occupation. Overall there was a significant (p < 0.001) relationship between the animal species kept and the main occupation of the keeper: crop/vegetable farmers tended to keep goats only or both sheep and goats, while people without any other income generating activity were more inclined to keep sheep only.

### Reasons for keeping small ruminants

The frequencies of primary reasons for keeping sheep and goats varied significantly (p < 0.001; Table 1) across locations. For both sheep and goats, the most frequently reported reason for keeping in Kano and Bobo Dioulasso was “Sale of live animals for cash in case of emergency” which refers to the insurance function of these livestock species, while “Herd size as social status and pleasure” followed by “Herd size as capital asset” were the two most frequently evoked reasons in Sikasso. Within a location, the importance given to different functions varied between species, with variations being significant (p < 0.05) in Kano and Sikasso. “Sale of live animals for extra cash during Eid al-Kabir” was more frequently reported for sheep than for goats in Kano, whereas in Sikasso “Sale of live animals for regular cash requirements” was more frequently reported for goats than for sheep (Table 2). The distribution of the ranks given by respondents to different functions within each location and for each species were significantly (p < 0.001) different. Within species, there were also significant differences across locations. “Sale of live animals for cash in case of emergency”, which refers to the insurance function of the animals, was given the highest rank for both sheep and goats by respondents in Kano and Bobo Dioulasso. In Sikasso it ranked only fourth in importance for sheep and fifth for goats while “Herd size as capital asset” was ranked first for both species. Within a location, there were also notable differences between species. While sheep were significantly (p < 0.001) less valued than goats for the security function, i.e. “Sale of live animals for cash to fulfill expected expenses” in Kano, they were significantly (p < 0.001) preferred to goats in Kano and Sikasso for “Extra cash during Eid al-Kabir*”.* In Sikasso, there was a significantly higher valuation of goats for “Sale for regular cash income” and for “Use for cultural/ritual purposes”.

**Table 1:** Frequencies (%) of primary functions of sheep and goats kept in the three West African cities of Kano (Nigeria), Bobo Dioulasso (Burkina Faso) and Sikasso (Mali)

Function	Sheep			Goats			Overall sheep (n = 301)	Overall goats (n = 306)
	Kano (n = 100)	Bobo Dioulasso (n = 102)	Sikasso (n = 99)	Kano (n = 100)	Bobo Dioulasso (n = 99)	Sikasso (n = 107)		
Sale of live animals for regular cash requirements	6.0	2.0	7.1	4.0	4.0	22.4	5.0	10.5
Sale of live animals for extra cash during Eid al-Kabir	22.0	1.0	0.0	8.0	2.0	0.0	7.6	3.3
Sale of live animals for cash to fulfill expected expenses	16.0	10.8	4.0	19.0	9.1	1.9	10.3	9.8
Sale of live animals for cash in case of emergency	29.0	48.0	8.1	54.0	65.7	0.9	28.6	39.2
Herd size as capital asset (storage of capital)	8.0	7.8	27.3	2.0	3.0	28.0	14.3	11.4
Use of animals for cultural/ritual/religious events	19.0	10.8	5.0	12.0	8.1	4.7	11.6	8.2
Herd size as social status and pleasure (prestige)	0.0	19.6	48.5	1.0	8.1	42.1	22.6	17.6
Statistics								
χ^2^	156.6			212.2			20.9	
*p*≤	0.001			0.001			0.002

**Table 2:** Comparative analysis of preference rankings of functions of sheep and goats in the three West African cities of Kano (Nigeria), Bobo Dioulasso (Burkina Faso) and Sikasso (Mali)

Function	Sheep						Goats					
	Mean rank^a^ (Rank)						Mean rank^a^ (Rank)					
	Kano (n = 100)		Bobo Dioulasso (n = 102)		Sikasso (n = 99)		Kano (n = 100)		Bobo Dioulasso (n = 99)		Sikasso (n = 107)	
Sale of live animals for regular cash income	4.74^aA^	(6)	5.32^aB^	(7)	4.88^acC**α**^	(7)	4.90^aA^	(6)	5.23^aA^	(6)	3.16^aB*β*^	(2)
Sale of live animals for extra cash during Eid al-Kabir	3.56^bA*α*^	(2)	4.99^aB^	(6)	3.39^bA**α**^	(2)	4.57^abA*β*^	(5)	5.24^aB^	(7)	5.10^bB*β*^	(7)
Sale of live animals for cash to fulfill expected expenses	3.65^bA*α*^	(3)	3.67^bA^	(3)	4.43^aA**α**^	(5)	2.93^cA*β*^	(2)	3.39^bdB^	(3)	4.93^bC*β*^	(6)
Sale of live animals for cash in case of emergency	2.47^cA*α*^	(1)	2.35^cA*α*^	(1)	4.15^cB**α**^	(4)	1.80^dA*β*^	(1)	1.77^cA**β**^	(1)	4.91^bB*β*^	(5)
Herd size as capital asset (storage of capital)	4.29^aA^	(5)	4.57^aA^	(5)	3.02^bB^	(1)	4.27^aeA^	(4)	4.97^afB^	(5)	3.03^aC^	(1)
Use of animals for cultural/ritual/religious events	3.85^abA^	(4)	3.32^bB^	(2)	4.64^acA*α*^	(6)	4.12^beA^	(3)	3.08^bB^	(2)	3.54^aB*β*^	(4)
Herd size as social status and pleasure (prestige)	5.46^dA^	(7)	3.79^bB*α*^	(4)	3.51^bC^	(3)	5.43^fA^	(7)	4.34^dfB*β*^	(4)	3.33^aC^	(3)

^a^Means in columns followed by different lower case letters are different at the Bonferroni-adjusted significance level p ≤ 0.002 (Friedman test followed by Wilcoxon signed-rank post hoc tests with Bonferroni’s correction for multiple comparisons); Means of the same species (sheep or goats) followed by different uppercase letters in rows are different at *p* ≤ 0.05 (Kruskall-Wallis test followed by Mann–Whitney U tests). Significant differences at p ≤ 0.01 (Mann–Whitney *U* test) between sheep and goats within an individual city are indicated by italic Greek letters.

Only 16 goat farmers (none in Kano, 8 in Bobo Dioulasso and 8 in Sikasso) and 4 sheep farmers (none in Kano, 3 in Bobo Dioulasso and 1 in Sikasso) were milking their animals and used the milk for household consumption, primarily for infant feeding (15 out of 20 respondents).

### Flock sizes and composition

The overall average flock size was 9.2 heads for sheep (ranging from 1 to 48 animals) and 11.5 heads for goats (ranging from 1 to 40 animals) with significant (p < 0.001) differences across cities (Table 3). The average size of sheep flocks was significantly (p < 0.001) higher in Kano than in Sikasso and Bobo Dioulasso, whereas the average size of goat flocks was significantly (p < 0.001) higher in Sikasso than in the two other cities. Furthermore, the average size of goat flocks was significantly (p < 0.001) higher than that of sheep in Sikasso, contrasting with results from Kano and Bobo Dioulasso. In general, female mature animals represented approximately 50% of the total flock and mature male animals accounted for 22 and 14% of sheep and goats flocks, respectively. However there were significant variations between cities and species. The proportion of mature (≥12 months) females in goat flocks was significantly (p < 0.05) higher in Sikasso than in Bobo Dioulasso and Kano. Compared to sheep flocks, it was also significantly higher (p < 0.05) in Sikasso and lower (p < 0.05) in Kano. The proportion of mature (≥12 months) males was significantly higher in sheep than in goat flocks irrespective of city. For both species, it was significantly higher in Kano than in Sikasso and Bobo Dioulasso (p < 0.05). The percentage of sheep and goat flocks without any mature male was significantly (p < 0.001) higher in Bobo Dioulasso (27 and 48%, respectively) than in Kano (6 and 24%) and Sikasso (9 and 6%). With the exception of Sikasso, the proportion of flocks without mature males was significantly (p < 0.001) higher for goats than for sheep.

**Table 3:** Average sheep and goat flock sizes (n) and flock composition (% of flock size) in the three West African cities of Kano (Nigeria), Bobo Dioulasso (Burkina Faso) and Sikasso (Mali)

Variable	Overall		Kano		Bobo Dioulasso		Sikasso	
	n	Mean ± SD	n	Mean^a^ ± SD	n	Mean^a^ ± SD	n	Mean^a^ ± SD
Flock size (*n*)								
Sheep	302	9.2 ± 6.0	100	10.7^aA^ ± 7.2	102	8.1^aBC^ ± 5.1	100	8.9^aC^ ± 5.2
Goats	307	11.5 ± 7.8	100	8.8^bA^ ± 6.4	100	7.8^aA^ ± 5.6	107	17.4^bB^ ± 7.3
Flock structure (%)								
Female mature (≥12 months)								
Sheep	302	50.1 ± 15.2	100	50.8^aA^ ± 14.2	102	49.9^aA^ ± 17.2	100	49.5^aA^ ± 14.8
Goats	307	49.7 ± 15.6	100	44.0 ^bA^ ± 13.3	100	49.8^aB^ ± 17.9	107	54.9^bC^ ± 13.5
Female young (<12 months)								
Sheep	302	16.2 ± 14.0	100	13.7^aA^ ± 11.6	102	14.4^aA^ ± 13.7	100	20.4^aB^ ± 15.6
Goats	307	21.0 ± 14.9	100	22.5^bA^ ± 15.8	100	21.8^bA^ ± 15.2	107	18.9^aA^ ± 13.7
Male mature (≥12 months)								
Sheep	302	21.7 ± 15.3	100	26.3^aA^ ± 12.1	102	17.8^aB^ ± 17.1	100	21.0^aB^ ± 12.1
Goats	307	14.3 ± 12.3	100	18.2^bA^ ± 13.5	100	9.4^bB^ ± 13.4	107	14.9^bC^ ± 8.3
Male young (<12 months)								
Sheep	302	11.7 ± 12.5	100	9.1^aA^ ± 10.7	102	17.5^aB^ ± 13.9	100	8.7^aA^ ± 10.7
Goats	307	13.2 ± 12.3	100	12.2^bA^ ± 11.7	100	18.6^aB^ ± 14.0	107	9.4^aA^ ± 9.5
Castrates								
Sheep	302	0.3 ± 2.1	100	0.2^aA^ ± 1.7	102	0.3^aA^ ± 2.1	100	0.3^aA^ ± 2.3
Goats	307	1.8 ± 5.1	100	3.1^bA^ ± 2.0	100	0.3^aB^ ± 2.0	107	1.8^bA^ ± 4.6

^a^Means in columns followed by different lowercase letters are different at p ≤ 0.05 (Mann–Whitney U test); Means in rows followed by different uppercase letters are different at p ≤ 0.05 (Kruskall Wallis test followed by Mann–Whitney U test).

### Breeding practices

#### Breeds

**Figure 1:**
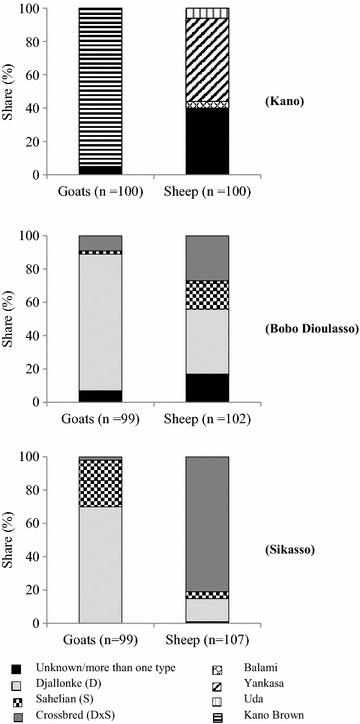
Distribution of animal genotypes among urban flocks of goats and sheep in Kano (Nigeria), Bobo Dioulasso (Burkina Faso) and Sikasso (Mali).

Irrespective of city, most goats were of the indigenous type. The Kano Brown goat was kept by 95% of respondents in Kano, whereas the Djallonke was predominant in Bobo Dioulasso (82%) and in Sikasso (70%). In contrast, keeping crossbred animals and/or maintaining more than one genotype in the same flock was more commonly practiced by sheep keepers in all three cities (Figure 1).

#### Management of breeding stock and selection criteria

The main sources of breeding stock varied significantly (p < 0.001) across cities. Respondents in Kano got their first breeding animals from rural markets (43.5%), followed by urban farms (25%) and gift/inheritance (14%), whereas those in Bobo Dioulasso and Sikasso consistently mentioned urban markets (36 and 50%, respectively) followed in Sikasso by rural markets (34%) and rural farms (13%), and in Bobo Dioulasso by rural farms (23%) and gift/inheritance (19%). Irrespective of city and species, undesired animals were culled. The main reasons for culling animals varied significantly (p < 0.001) between cities and between species within cities. The three main reasons for culling both sheep and goats were poor health condition (92 and 90%, respectively, in Kano; 55% for both species in Bobo Dioulasso; 91 and 73% in Sikasso), old age (69 and 66% in Kano; 72 and 71% in Bobo Dioulasso; 88 and 46% in Sikasso) and poor fertility (66 and 74% in Kano; 44 and 53% in Bobo Dioulasso; 99 and 84% in Sikasso).

**Figure 2:**
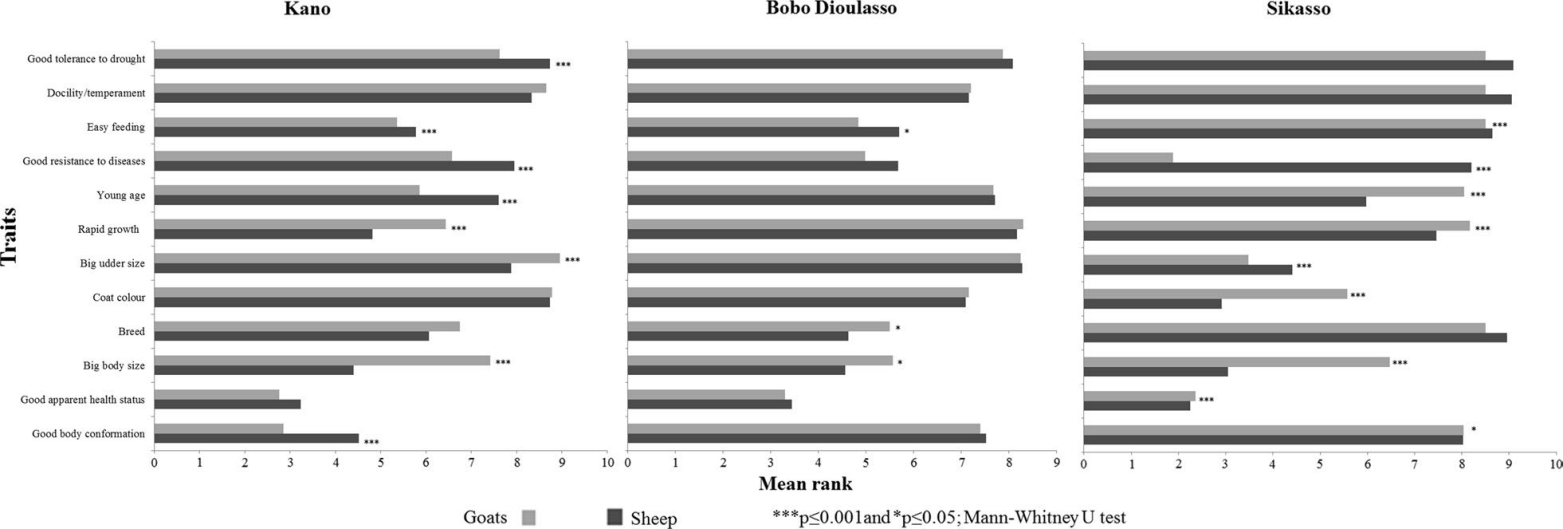
Between species comparison of preference rankings of individual traits used to select breeding females in the three West African cities of Kano (Nigeria), Bobo Dioulasso (Burkina Faso) and Sikasso (Mali).

**Figure 3:**
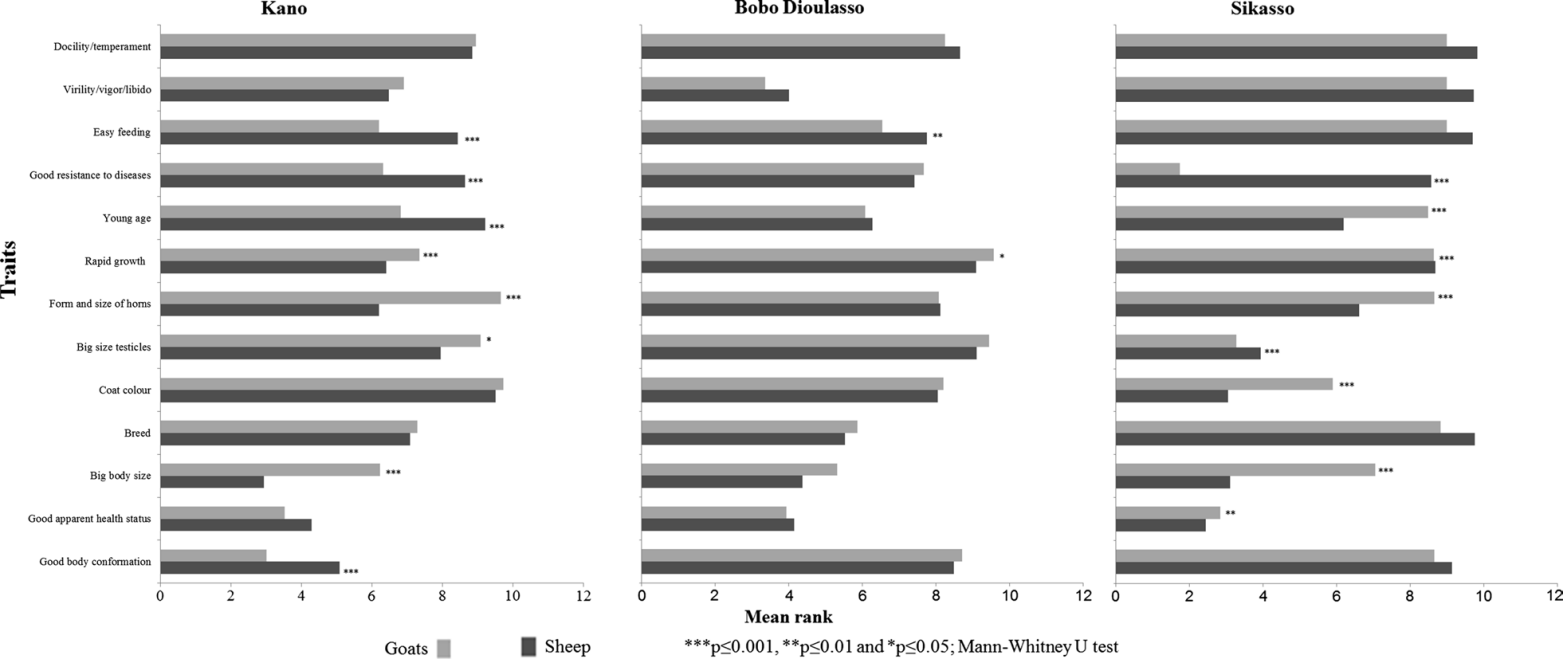
Between species comparison of preference rankings of individual traits used to select replacing breeding males in the three West African cities of Kano (Nigeria), Bobo Dioulasso (Burkina Faso) and Sikasso (Mali).

Irrespective of city and species, all replacing breeding females originated from within the respective flock. Replacement of breeding males from their own flock was practiced by a significantly (p < 0.001) higher proportion of farmers in Sikasso (97 and 100% for sheep and goats, respectively) than in Bobo Dioulasso (70 and 77%) and in Kano (48 and 73%). When selecting a female for replacement, sheep and goat keepers considered both the individual’s own characteristics and the characteristics of the individual’s mother (all sheep and goat keepers in Sikasso against 97 and 92%, respectively, in Kano, and 89 and 95%, respectively, in Bobo Dioulasso). Criteria applied when selecting breeding females on the basis of their own characteristics are summarized in Table 4. In all locations and irrespective of species, the apparent health status of the animal was ranked first or second. For both species, general body conformation was significantly (p < 0.001) more important in Kano than in the two other cities. Coat color, which is an esthetic characteristic, was only ranked high in Sikasso, whereas breed was seen as an important characteristic only in Bobo Dioulasso. Temperament and tolerance to drought were also mentioned as selection criteria, but were ranked relatively low in all cities and for both species. Within city, there were also significant differences between species for the importance attached to some criteria (Figure 2). In all three cities, big body size was ranked significantly (p < 0.05) higher for sheep than for goats. Similarly, in Sikasso and Kano, resistance to diseases/parasites was ranked significantly (p < 0.001) higher for goats than for sheep. Mother’s fertility was the most frequently reported trait for sheep in all three locations (93% in Kano, 58.8% in Bobo Dioulasso and 94% in Sikasso) and for goats in Kano (90%) and Bobo Dioulasso (67%), whereas the large majority of goats farmers (92%) in Sikasso selected on the basis of the mother’s ability to resist to diseases and parasites. Irrespective of city, this trait was mentioned by significantly (p < 0.001) higher proportions of goat farmers (64% in Kano, 52% in Bobo Dioulasso and 92% in Sikasso) than of sheep keepers (30% in Kano, 45% in Bobo Dioulasso and only 17% in Sikasso). Mother’s prolificacy was equally important for selecting sheep and goats for breeding purposes in Kano, and was the second most reported trait after mother’s fertility. It was mentioned by significantly (p < 0.001) higher proportions of goat than of sheep farmers in the two other cities (61% against 35% of sheep farmers in Bobo Dioulasso, and 77% against 43% in Sikasso). Mother’s docility/temperament was also mentioned in Kano (18% of sheep and 23% of goat farmers) and Bobo Dioulasso (39% of sheep and 29% of goat farmers), whereas its breed and coat color were mentioned in Kano only by few respondents (11% of sheep farmers for both traits against 13% and 6% of goat farmers, respectively, for mother’s breed and coat colour). Irrespective of city and species, the majority of respondents (97%) reported that they do select males for breeding purposes. Ranking of criteria used to select replacing rams and bucks varied significantly (p < 0.05) across cities (Table 5). Most important criteria of sheep farmers were related to physical appearance: Big body size ranked first in Kano, followed by good apparent health and body conformation. High libido, which is a behavioral trait, ranked highest in Bobo Dioulasso, followed by good apparent health status and body size, whereas in Sikasso good apparent health status ranked first and was followed by coat color and body size. Goat farmers reported highest preference for good body conformation in Kano, for high libido in Bobo Dioulasso and for resistance to disease and parasites in Sikasso. With the exception of Bobo Dioulasso, ranks attributed to most preferred traits significantly differed (p < 0.05) between both species (Figure 3). Irrespective of species, coat colour was ranked high in Sikasso, but was unimportant in Kano and Bobo Dioulasso. Similarly, breed was important in Bobo Dioulasso, but unimportant in Kano and Sikasso.

**Table 4:** Comparative analysis of preference rankings of individual traits of female animals used as selection criteria across the three West African cities of Kano (Nigeria), Bobo Dioulasso (Burkina Faso) and Sikasso (Mali)

Traits	Sheep						Goats					
	Mean rank^a^ (Rank)						Mean rank^a^ (Rank)					
	Kano (n = 100)		Bobo Dioulasso (n = 102)		Sikasso (n = 99)		Kano (n = 100)		Bobo Dioulasso (n = 99)		Sikasso (n = 107)	
Good body conformation	4.51^aA^	(3)	7.52^Bfad^	(8)	8.02^Baf^	(7)	2.85^aA^	(2)	7.40^Bad^	(8)	8.04^Ca^	(6)
Good apparent health status	3.23^bA^	(1)	3.44^Ab^	(1)	2.25^Ab^	(1)	2.76^aA^	(1)	3.30^Ab^	(1)	2.36^Ab^	(2)
Big body size	4.40^Aa^	(2)	4.56^Ac^	(2)	3.05^Bbc^	(3)	7.42^bgA^	(8)	5.56^Bc^	(5)	6.47^Ac^	(5)
Breed	6.06^Ac^	(6)	4.63^Bc^	(3)	8.96^Cdi^	(10)	6.75^bcgA^	(7)	5.50^Bc^	(4)	8.50^Ad^	(9)
Coat colour	8.74^Ad^	(11)	7.09^Bd^	(6)	2.92^Cc^	(2)	8.78^dA^	(11)	7.16^Ba^	(6)	5.57^Ce^	(4)
Big udder size	7.88^Ae^	(8)	8.28^Be^	(12)	4.41^Ce^	(4)	8.96^dA^	(12)	8.24^Ae^	(11)	3.48^Bf^	(3)
Rapid growth	4.82^Aa^	(4)	8.17^Baef^	(11)	7.46^Cf^	(6)	6.44^ceA^	(5)	8.30^Be^	(12)	8.17^Ca^	(8)
Young age	7.60^Ae^	(7)	7.71^Afg^	(9)	5.97^Bg^	(5)	5.86^efA^	(4)	7.67^Bda^	(9)	8.05^Ba^	(7)
Resistance to disease	7.95^Ae^	(9)	5.67^Bh^	(4)	8.20^Aah^	(8)	6.57^bcA^	(6)	4.98^Bc^	(3)	1.88^Cg^	(1)
Easy feeding	5.77^Ac^	(5)	5.69^Ah^	(5)	8.64^Bdh^	(9)	5.36^fA^	(3)	4.84^Ac^	(2)	8.50^Bd^	(10)
Docility/temperament	8.33^Ad^	(10)	7.16^Bd^	(7)	9.06 ^Ci^	(11)	8.65^dA^	(10)	7.20^Bfa^	(7)	8.50^Cd^	(11)
Tolerance to drought	8.74^Ad^	(12)	8.08^Aeg^	(10)	9.09^Bi^	(12)	7.63^gA^	(9)	7.87^Bed^	(10)	8.50^Cd^	(12)

^a^Means in columns followed by different lowercase letters are different at the Bonferroni-adjusted significance level p ≤ 0.002 (Friedman test followed by Wilcoxon signed-rank post hoc tests with Bonferroni’s correction for multiple comparisons); Means of the same species (sheep or goat) followed by different uppercase letters in rows are different at p ≤ 0.05 (Kruskall Wallis test followed by Mann–Whitney U tests).

**Table 5:** Comparative analysis of preference rankings of individual traits of male animals used as selection criteria across the three West African cities of Kano (Nigeria), Bobo Dioulasso (Burkina Faso) and Sikasso (Mali)

Traits	Sheep						Goats					
	Mean rank^a^ (Rank)						Mean rank^a^ (Rank)					
	Kano (n = 100)		Bobo Dioulasso (n = 98)		Sikasso (n = 99)		Kano (n = 100)		Bobo Dioulasso (n = 99)		Sikasso (n = 107)	
Good body conformation	5.08^a^	(3)	8.49^b^	(10)	9.13^b^	(9)	3.01^a^	(1)	8.71^b^	(11)	8.66^b^	(8)
Good apparent health status	4.28^a^	(2)	4.14^a^	(2)	2.44^c^	(1)	3.52^a b^	(2)	3.93^a^	(2)	2.83^b^	(2)
Big body size	2.94^a^	(1)	4.37^b^	(3)	3.10^a^	(3)	6.23^a^	(4)	5.31^a^	(3)	7.05^b^	(5)
Breed	7.08^a^	(7)	5.52^b^	(4)	9.75^c^	(12)	7.28^a^	(8)	5.87^b^	(4)	8.82^c^	(10)
Coat colour	9.50^a^	(13)	8.05^b^	(8)	3.04^c^	(2)	9.72^a^	(13)	8.21^b^	(9)	5.89^c^	(4)
Big size testicles	7.95^a^	(8)	9.11^b^	(13)	3.94^c^	(4)	9.08^a^	(11)	9.45^b^	(12)	3.27^c^	(3)
Form and size of horns	6.19^a^	(4)	8.12^b^	(9)	6.61^a^	(6)	9.65^a^	(12)	8.08^b^	(8)	8.66^a^	(9)
Rapid growth	6.40^a^	(5)	9.09^b^	(12)	8.69^c^	(8)	7.35^a^	(9)	9.57^b^	(13)	8.64^c^	(7)
Young age	9.21^a^	(12)	6.28^b^	(5)	6.19^b^	(5)	6.82^a^	(6)	6.07^a^	(5)	8.49^b^	(6)
Resistance to disease	8.64^a^	(10)	7.41^b^	(6)	8.57^a^	(7)	6.32^a^	(5)	7.67^b^	(7)	1.73^c^	(1)
Easy feeding	8.43^a^	(9)	7.76a	(7)	9.70^c^	(10)	6.19^a^	(3)	6.54^a^	(6)	8.99^b^	(11)
Virility/vigor/libido	6.47^a^	(6)	4.01^b^	(1)	9.73^c^	(11)	6.91^a^	(7)	3.36^b^	(1)	8.99^b^	(12)
Docility/temperament	8.85^a^	(11)	8.65a	(11)	9.83^c^	(13)	8.94^a^	(10)	8.24^a^	(10)	8.99^b^	(13)

^a^Means in columns followed by different lowercase letters are different at the Bonferroni-adjusted significance level p ≤ 0.002 (Friedman test followed by Wilcoxon signed-rank post hoc tests with Bonferroni’s correction for multiple comparisons).

#### Practices of castration

In both sheep and goat flocks, breeding males originated from the same respective flocks. Castration of male sheep was an uncommon practice in the three cities. It was not mentioned at all in Kano, and was reported by only 5% and 1% of sheep keepers in Bobo Dioulasso and Sikasso, respectively. In contrast, castration of male goats was more commonly practiced and was significantly (p < 0.001) more frequent in Kano (54% of respondents) than in Bobo Dioulasso (22%) and Sikasso (18%); main reasons for castration were to eliminate the “buck odor” and to improve growth rates. Age at castration ranged from 2 weeks to 2 years with an average age of 7.6 months in Kano, 8.4 months in Bobo Dioulasso and 7.0 months in Sikasso.

## Discussion

While a previous paper (Amadou et al. 2012) analyzed and compared the opportunities, constraints and feeding strategies of small ruminants across the three selected cities using a systems approach, this paper concentrates on and compares production objectives and breeding practices.

### Production objectives

Our results reveal the multi-functionality of goats and sheep among urban dwellers in the three locations investigated, whereby their financial functions were ranked as of paramount importance. This finding is consistent with what was stated in earlier studies by Touré and Ouattara (2001), Ajala et al. (2008) and Baah et al. (2012) in selected urban communities in Côte d’Ivoire, Nigeria and Ghana, respectively, but contrasts with reports by Thys and Ekembe (1992) and Lawal-Adebowale (2012) who described that urban small ruminants are mainly raised for home consumption. The remarkable variations of the production objectives across locations and between species observed in the current study are likely to be related to differences in social and economic contexts. For example, the objective “Sale of live animals for extra cash during Eid al-Kabir” for sheep production was of significantly higher importance in Kano and Sikasso compared to Bobo Dioulasso, probably because of the higher proportion of Muslims and thus higher demand for sheep in these cities during this Islamic holiday. Milk production was no production objective for urban small ruminants; this has been confirmed by the very insignificant number of goat and sheep keepers who were milking their animals and can be explained by cultural preferences for goat and sheep meat and bias against the consumption of their milk in the study locations. Consumption of goat milk is even regarded as taboo in many West African societies (Belewu and Aiyegbusi 2002; Dubeuf 2010). The lack of awareness of its nutritional advantages is probably a further explanation of the unpopularity of small ruminant milk in the study area.

The majority of goat and sheep farmers were engaged full-time in other income-generating activities, indicating that small ruminant keeping represented a secondary activity. The remarkably higher proportion of people without any income-generating activity engaged in sheep production compared to goat production suggests that sheep are perceived as of higher economic value than goats. Conversely, the higher proportion of goat than of sheep farmers engaged in crop farming/gardening as primary activity may be indicative of a higher potential of this species to valorize highly-fibrous crop residues (Aregheore 1996) as far as integration to urban crop farming and gardening is concerned. It is noteworthy that urban small ruminant owners were predominantly male people. While this observation agrees with recent reports from Ghana (Baah et al. 2012), Nigeria (Lawal-Adebowale 2012) and Kenya (Kagira and Kanyari 2010), it sharply contrasts with results from rural areas in West Africa, where small ruminant keeping was found to be closely associated with women (Jaitner et al. 2001; Dossa et al. 2008; Smith et al. 2013). This finding, together with the financial functions of the animals, reflects the transformation of urban small ruminant keeping from a subsistence activity associated with women to a commercially viable enterprise in which men dominate—similar to observations made with respect to the urban gardening sector in Bobo Dioulasso (Freidberg 2001) and Bamako (Wooten 2003). The average age of small ruminant owners was 44 years indicating that urban small ruminant keepers were mainly people of middle age. The significantly older age of small ruminant owners in Bobo Dioulasso and Sikasso compared to Kano is indicative of a higher potential for improved management practices in Kano given that young people are generally more receptive to innovations and new technologies than old ones (Adesina et al. 2000). Furthermore, by considering the important functions of insurance and economic security of small ruminants, the relative young age of their owners irrespective of city, and the important number amongst them who started this activity in the last 10 years (73% of the total number of small ruminant keepers included in this study), we argue that this activity will continue to flourish in the surveyed cities and elsewhere in urban areas of West Africa.

### Breeding practices

Taking into consideration the small flock sizes, the low practice of castration, especially among sheep farmers, and the complete lack of keeping breeding records, the selection of replacement animals from within the flock as observed among the surveyed farmers represents a high risk of inbreeding of which they were probably not aware. The implication of this for the development of more viable urban sheep and goat enterprises is to create awareness among farmers (1) on the negative effects of inbreeding and (2) on the importance of individual identification of animals and breeding record keeping in their farm management strategies, and (3) to provide them with an appropriate easy-to-use paper-based or/and mobile phone/smartphone-based record keeping system, as these electronic devices are in widespread use among urban dwellers in Sub Saharan Africa (Porter 2015). As revealed by the results of the current study, irrespective of city and species, replacement animals were selected mainly on the basis of their general appearance and condition. Record keeping will enable farmers to shift from the current practice to a selection of better performing animals for replacement, based on pedigree information and breeding performances. The higher proportions of mature male sheep compared to goats could probably be explained by the low practice of castration among sheep farmers irrespective of city, and this is due to the fact that a ram for “Eid al-Kabir” (the Islamic festival of sacrifice) needs to be intact, i.e. not castrated. The complete lack of breeding males in several sheep and goat flocks in Bobo Dioulasso compared to the other two cities might result in delayed services and negatively affect the flocks’ reproductive performance and overall productivity.

### Preference for selection traits and implications for future breeding programs

Both sheep and goats seemed to be selected based on the same individual physical traits in all three locations. However, the emphasis put on each trait varied across cities and between species, reflecting differences in production objectives as mentioned above. Of particular note is the higher emphasis put on coat color in Sikasso compared to Bobo Dioulasso and Kano. Although certain beliefs regarding raising and/or eating meat of animals of certain coat colour are widespread across many West African societies (Brisebarre and Kuczynski 2009) and elsewhere in Africa (Gwaze et al. 2009), they were remarkably more pronounced among respondents in Sikasso compared to the two other cities and could be explained by cultural differences. The integration of animals into the traditional beliefs was not quantified in the current study because most respondents avoided mentioning this aspect. When selecting female replacement animals on the basis of their mother’s characteristics, farmers showed a strong preference for productivity traits such as fertility and prolificacy irrespective of species and city. This reflects the farmers’ general objective which is to increase flock size and overall flock productivity. It is noteworthy that the mother’s ability to resist diseases and parasites, which is an adaptive trait, was more frequently reported and ranked higher by goat than by sheep farmers. This is probably related to higher mortality rates in goats than in sheep, especially in Sikasso. Although, with the exception of Bobo Dioulasso, breed was ranked least by both sheep and goat farmers, a considerable high proportion of sheep farmers (Figure 1) tended to keep animals of improved genotypes, mainly crossbred, in Sikasso and Bobo Dioulasso; alternatively several genotypes were kept in the same flock (Kano and Bobo Dioulasso). In contrast, goats were mostly of local genotypes irrespective of city. This remarkable higher motivation of sheep farmers for improved breeding in the three cities is likely to reflect the market and non-market preferences for non-local breeds of sheep which are influenced by both culture and religion (Brisebarre and Kuczynski 2009). For “Eid al-Kabir”, rams with special traits are preferred and long-legged sheep types such as the Yankasa sheep in Kano and the Sahelian sheep in Bobo Dioulasso and Sikasso fit these preferences well. White rams (Ayantunde et al. 2007), especially of the Sahelian Bali–Bali breed, and/or their crossbreds with Djallonke (Brisebarre and Kuczynski 2009) are preferred for “Eid al-Kabir” due to their size and heavier body weight and fetch higher market prices. Overall, these findings provide evidence that preferential selection of breeding animals is a common practice among both urban sheep and goat farmers, and suggest that there is less selection pressure on goats than on sheep, mainly because of the higher cultural and economic values of the latter. This leads to the hypothesis that irrespective of the city urban sheep farmers would be more interested than goat keepers to adopt improved breeding practices and to participate in the development and implementation of carefully designed genetic and management improvement programs.

## Conclusions

Sheep and goats have multipurpose functions for the livelihoods of urban people in West Africa, even though they are predominantly kept for financial and cultural reasons. In contrast to rural production, urban small ruminant keeping is dominated by men. The variations in the relative importance of different functions and preferences for specific traits across cities and species reflect socio-economic and cultural differences. The study results suggest that improvement of daily handling and breeding is economically more desirable for urban sheep farmers than for urban goat keepers. There is thus a need to assist the former in their upgrading efforts by engaging them in the design and establishment of sustainable management and genetic improvement programs.
